# Adapting an Osteoarthritis Peer Mentorship Intervention for Remote Delivery to People Experiencing Socioeconomic Disadvantage: A Multi‐Method Approach

**DOI:** 10.1111/hex.70245

**Published:** 2025-04-01

**Authors:** Anna M. Anderson, Elizabeth Lavender, Samantha Mason, Linda Eckersley, Susan Barry, Amrit Daffu‐O'Reilly, Heidi Green, Mark Conner, Gretl A. McHugh

**Affiliations:** ^1^ School of Healthcare University of Leeds Leeds UK; ^2^ Leeds Institute of Health Sciences University of Leeds Leeds UK; ^3^ Leeds Institute of Rheumatic and Musculoskeletal Medicine University of Leeds Leeds UK; ^4^ National Institute for Health and Care Research (NIHR) HealthTech Research Centre in Accelerated Surgical Care Leeds UK; ^5^ COUCH Health Manchester UK; ^6^ School of Psychology University of Leeds Leeds UK

**Keywords:** intervention adaptation, osteoarthritis, peer mentorship, public involvement, self‐management, socioeconomic disadvantage

## Abstract

**Background:**

Osteoarthritis (OA) is a common musculoskeletal condition which can cause debilitating pain and other symptoms. OA is more prevalent, and the impact is greater, among people experiencing socioeconomic disadvantage. While peer support is a recommended strategy for addressing these health inequalities, evidence in this area is limited. We previously developed and feasibility tested an in‐person OA peer mentorship intervention in a group with limited diversity. This study adapted the intervention for remote delivery to people experiencing socioeconomic disadvantage.

**Methods:**

This multi‐method study was informed by the ADAPT guidance. Focus groups and interviews were conducted with 20 adults with hip/knee OA experiencing socioeconomic disadvantage to explore barriers and enablers to engagement with remote OA peer mentorship. The findings and project team members' suggestions informed provisional adaptations. The intervention was further adapted and finalised through two participatory workshops conducted with five people with relevant lived experience, four community organisation representatives, and six Patient and Public Involvement (PPI) representatives; and four intervention delivery practice runs undertaken by four PPI representatives.

**Findings:**

A wide range of barriers and enablers were identified to two target behaviours – using self‐management strategies and attending remote OA peer mentorship sessions. The identified barriers/enablers and additional study activities led to various adaptations. These spanned the delivery and content of the peer mentor training, mentorship sessions, and supporting resources. The adapted intervention consists of six 1‐h self‐management support sessions delivered remotely by a trained peer mentor. The remote format is flexible, with support available for addressing barriers related to making videoconferencing calls.

**Conclusions:**

This study rigorously and systematically adapted an in‐person OA peer mentorship intervention for remote delivery to people experiencing socioeconomic disadvantage. Employing a multi‐method approach with diverse partners was key to identifying what adaptations were required.

**Patient or Public Contribution:**

PPI representatives played a central role in this study as project team members (two individuals), Project Advisory Group members (three individuals), and wider PPI group members (six additional individuals). This extensive PPI aimed to ensure the adapted OA peer mentorship intervention is useful, acceptable, and accessible to the people it aims to benefit.

**Trial Registration:**

ISRCTN registration of the overall project was obtained on 18 May 2023 (ISRCTN78088278).

## 1 Introduction

Osteoarthritis (OA) is a long‐term musculoskeletal (MSK) condition mainly affecting older adults [1]. Approximately 10 million people have OA in the United Kingdom (UK) alone, with the hip and knee being the most common sites [1]. While some people with OA are minimally affected, others experience debilitating symptoms such as joint pain, stiffness, and mobility limitations [1, 2]. These symptoms can profoundly affect people's daily activities, ability to work, and mental wellbeing [1]. OA is also associated with social isolation and numerous other long‐term conditions and generates large costs for individuals and wider society [1, 3].

OA is more prevalent, and the impact is greater, among people experiencing socioeconomic disadvantage [4]. While there is no widely accepted definition of socioeconomic disadvantage, it is generally used to refer to people *‘*living in less favourable social and economic circumstances than the majority of others in the same society*’* [5, 6]. Many indicators of socioeconomic disadvantage have been identified, such as living in a deprived area, having low educational attainment, being unemployed, and being a refugee [5, 6].

People experiencing socioeconomic disadvantage may face a range of barriers to accessing formal healthcare such as social distance from health professionals, transportation issues, and competing priorities [7, 8, 9, 10]. These and other barriers may limit people's engagement with self‐management strategies, which are widely recognised as key to managing OA [8, 11]. For example, people experiencing socioeconomic disadvantage may lack social support to exercise or find it difficult to eat a healthy diet due to food insecurity [8, 11]. Engagement with self‐management interventions is lower among people experiencing socioeconomic disadvantage; hence, self‐management interventions may increase health inequalities if they are not appropriately tailored to the needs of low socioeconomic groups [12].

The UK Arthritis and Musculoskeletal Alliance (ARMA) recently published a report on tackling MSK health inequalities [8]. This recommends providing supported self‐management strategies, including peer support. A systematic review and meta‐analysis suggested peer support interventions for adults with chronic MSK pain may improve pain, self‐efficacy and function compared to usual care, but the evidence certainty was low to very low [13]. This review highlighted few studies investigated one‐to‐one peer support, despite the benefits of tailoring support to people's individual needs. Additionally, few studies investigated online or telephone delivery formats. The authors reported this is likely to have limited recruitment of individuals from under‐served groups and suggested further research of telehealth‐based peer support interventions is warranted given their potential accessibility and scalability [13].

We previously developed a peer mentorship intervention in which trained volunteers with hip/knee OA (‘peer mentors’) delivered up to eight one‐to‐one community‐based self‐management support sessions to other people with OA (‘mentees’) [14]. Our feasibility study demonstrated the intervention was acceptable and feasible and may offer benefits for both peer mentors and mentees [14, 15, 16]. However, the mentors and mentees lacked diversity, and the intervention was only delivered in person. To help address these limitations and the inequalities highlighted above, we aimed to adapt our in‐person OA peer mentorship intervention for remote delivery to people experiencing socioeconomic disadvantage.

## 2 Methods

### 2.1 Design

**Figure 1:**
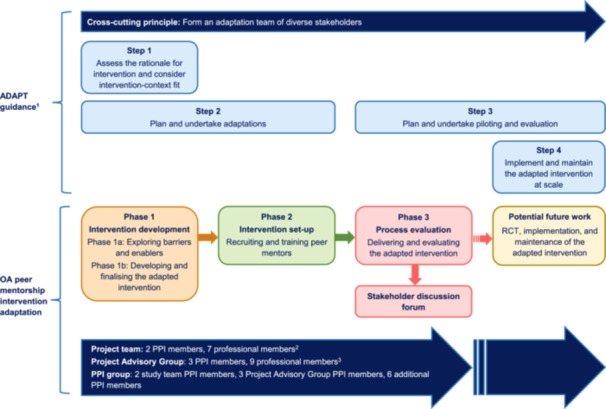
Project flow chart. ^1^The cross‐cutting principle and steps are quoted from the ADAPT guidance [17]. ^2^ The professional project team members included six university‐based researchers and a Director of Health Equity. ^3^The Project Advisory Group professional members included an independent chair, a community organisation representative, four clinical and academic experts, one coproduction and community engagement expert, and two project funder representatives. An image description of the flow chart is available in Supporting File S1. OA, osteoarthritis; PPI, patient and public involvement; RCT, randomised controlled trial.

This study is Phase 1 of a mixed‐methods project informed by the ADAPT guidance [17]. The subsequent phases will be reported elsewhere. This study addressed the ADAPT cross‐cutting principle and Steps 1 and 2 (Figure 1; Supporting File S1).

This study involved two subphases. Phase 1a involved identifying provisional adaptations for the OA peer mentorship intervention. Phase 1b involved developing and finalising the adapted intervention.

### 2.2 Theoretical Framework

To ensure the adaption process had a sound theoretical basis, this study drew on the Theoretical Domains Framework (TDF) [18, 19]. A key step when using the TDF is to specify the target behaviour(s). The OA peer mentorship intervention is a complex intervention targeting multiple behaviours. To ensure this study could be completed with the time/resources available, two broad target behaviours were specified:

1. Using self‐management strategies
2. Attending remote OA peer mentorship sessions

The TDF has been linked to the Capability Opportunity Motivation model of Behaviour (COM‐B) [20]. The COM‐B model was used in this study to structure the findings about influences on the target behaviours.

### 2.3 Ethical Approval, Registration and Reporting

Ethical approval for the overall project, including this study, was obtained from the South Birmingham Research Ethics Committee (23/WM/0108). All research participants provided written or electronic informed consent before participating. The overall project was registered on ISRCTN on 18 May 2023 (ISRCTN78088278). The study reporting has been informed by the ADAPT guidance [17], Framework for Reporting Adaptations and Modifications‐Enhanced (FRAME) [21], and GRIPP2 short form [22].

### 2.4 Project Team and Oversight

The overall project team includes two PPI members (L.E., S.B.) and seven professional members (Figure 1). Five members were involved in the previous feasibility study as peer mentors (L.E., S.B.), the project lead (G.A.M.), the Volunteer Coordinator (E.L.) or a Research Associate (A.M.A.). The project team's expertise spans various relevant areas, such as OA self‐management, psychology, and working with under‐served groups.

The overall project is being overseen by a Project Advisory Group (PAG) involving three PPI members and nine professional members (Figure 1). The PAG met once before, once during, and twice after this study, with one further meeting planned. Key PAG roles in this study included offering advice on recruitment‐related challenges, monitoring the study progress, and feeding back on the preliminary findings.

### 2.5 Patient and Public Involvement

The aim of PPI in the overall project is to ensure that the adapted OA peer mentorship intervention is useful, acceptable, and accessible to the people it aims to benefit. To help achieve this, PPI representatives are valued members of the project team and PAG (described above) and six additional PPI representatives are actively involved in a wider PPI group. All the wider PPI group members have hip and/or knee OA and direct or indirect experience of socioeconomic disadvantage. The wider PPI group met once before, once during, and twice after this study, with one further meeting planned. Key roles of the wider PPI group in this study included advising on the study plans, helping draft/revise public‐facing study materials, and helping to develop and finalise the adapted OA peer mentorship intervention as described below.

### 2.6 Phase 1a: Exploring Barriers and Enablers

#### 2.6.1 Overview

Phase 1a was a qualitative descriptive study focused on exploring barriers and enablers to engagement with a remote OA peer mentorship intervention among people experiencing socioeconomic disadvantage. The findings were used to inform provisional adaptations to the in‐person OA peer mentorship intervention.

#### 2.6.2 Participants

To facilitate recruitment of diverse participants, advertisements were shared via General Practices, social media, community organisations, PPI representatives, additional networks, and Egality – a community engagement agency dedicated to reducing health inequalities by improving inclusion in research. Recruitment strategies included targeting to neighbourhoods with high socioeconomic deprivation (20% most deprived) using the English Index of Multiple Deprivation (IMD) [23]. The advertisements included the wording ‘feel that you are disadvantaged because of your finances, education or social circumstances*’* rather than referring to socioeconomic disadvantage to promote understanding.

Adults able to provide informed consent were eligible if they:

- Had been diagnosed with hip/knee OA by a health professional and,
- Considered themselves to be experiencing socioeconomic disadvantage assessed by the question *‘*Do you consider yourself to be experiencing socioeconomic disadvantage?*’*

If a potential participant had difficulty understanding the socioeconomic disadvantage question, it was rephrased to ask if they felt they missed out on opportunities due to their financial, educational or social circumstances and examples were provided (Supporting File S2).

The socioeconomic disadvantage question was based on one of the project team member's previous projects [5], which highlighted that patients and the public may prefer terminology that emphasises the dynamic nature and potential transience of socioeconomic disadvantage. Asking a broad question about socioeconomic disadvantage was considered more appropriate than asking questions about specific indicators (e.g., income or educational attainment) given specific questions may have appeared more intrusive. Additionally, asking potential participants to self‐identify as experiencing socioeconomic disadvantage was considered preferable to relying on an area‐based deprivation index such as the English IMD as deprivation indices have conceptual and practical limitations [24]. For example, they are constructed based on subjective judgements (e.g., regarding the weighting of individual indicators/domains), and living in a deprived area does not necessarily mean someone is deprived and vice versa [24].

Maximum variation purposive sampling was employed based on joint affected (hip/knee), age, gender, and ethnicity [25, 26]. Individuals who saw the advertisements themselves or heard about the study via word‐of‐mouth were screened. During the recruitment, numerous contacts were received from suspected *‘*imposters*’* (individuals providing false identities [27, 28]). Table 1 summarises indicators of suspected imposters and strategies used to mitigate their participation.

**Table 1:** Indicators of suspected imposters and mitigating strategies.

Indicators of suspected imposters	Mitigating strategies
Batches of similar emails were received during a short space of time, including during the night.	The timing of emails was reviewed.
Numerous emails followed a similarly worded template, which was either very brief or used excessively formal language, sometimes with replication of wording from the study advertisements.	The wording of emails was reviewed.
Other aspects of the emails were concerning, such as one email including a survey intended for another organisation and one individual using several different email addresses.	The email addresses, email content, and any attachments were reviewed.
Individuals were difficult to communicate with, did not attend screening meetings, and/or were reluctant to turn their camera on.	Individuals were asked to join a screening meeting via telephone or videocall with a researcher as part of the recruitment process and were asked to turn their camera on where possible.
Individuals provided vague responses about where they heard about the study.	Individuals were asked where they heard about the study as part of the recruitment process.
Individuals used different names/aliases, often with a first name and surname that could both have been either a first name or surname, with the first names and surnames occasionally switched round.	Names of individuals were reviewed and checked for consistency.

There is no universally accepted approach for determining the sample size in qualitative studies, with approaches such as saturation, information power, and rules of thumb all receiving criticisms [29, 30]. This study involved reflexive thematic analysis as described by Braun and Clarke [31, 32], who suggest estimating an approximate sample size a priori and determining the final sample size during the data collection phase [29]. Correspondingly, it was estimated a priori that approximately 15 participants would be required [33, 34]. The final sample size of 20 participants was determined during the data collection phase to ensure sufficiently rich and diverse perspectives were obtained for answering the research question.

#### 2.6.3 Data Collection

The Phase 1a data collection was undertaken between July and September 2023. Data were primarily collected via focus groups to enable discussions between participants. To promote inclusion, participants were offered the opportunity to participate in a one‐to‐one interview if preferred. Based on the participants' preferences, two focus groups were held online, one focus group was held in person, four interviews were held via telephone, and one interview was held online with a family member present as a Patwari interpreter. All three focus groups included five participants. The online data collection was undertaken using Microsoft Teams. Participants were offered the opportunity to join a practice online meeting and receive training on using Microsoft Teams. Participants were offered a £25 recognition payment and reimbursement for WiFi costs as applicable.

Before their focus group/interview, participants completed a brief sociodemographic and clinical characteristics questionnaire administered via telephone by one researcher (A.D.O.). The focus groups were co‐facilitated by two researchers experienced in qualitative methods (E.L., A.D.O.). Two researchers conducted the individual interviews (E.L., A.D.O.).

The focus groups/interviews explored barriers and enablers to the target behaviours. The discussions were guided by a topic guide developed with reference to the TDF [18, 19]. The prompt questions covered self‐management, information needs, peer mentorship, and experience of using the Internet/videoconferencing (Supporting File S3). The topic guide was reviewed by both project team PPI members, who did not suggest any changes but provided advice on how to sensitively approach the topic areas. Due to the ethics application timing, pilot testing the topic guide was not feasible.

Field notes were recorded after the focus groups/interviews. The online focus groups and interview were recorded and automatically transcribed using the built‐in functionality of Microsoft Teams. The in‐person focus group and four telephone interviews were recorded using an encrypted digital recorder and transcribed by a professional transcription company.

#### 2.6.4 Data Analysis

Data were analysed using reflexive thematic analysis [31, 32]. QSR International NVivo software (version 14) and Microsoft Excel were used to help organise the data. All four researchers (A.M.A., E.L., S.M., A.D.O.) directly involved in the analysis employed reflexive approaches, such as recording and discussing reflections on the data collection and/or analysis process.

The transcripts were initially coded inductively by two researchers, with one (A.D.O.) coding all the transcripts and the other (E.L.) coding three transcripts. Three researchers (A.M.A., S.M., E.L.) reviewed the initial inductive coding to identify barriers and enablers to the target behaviours. The barriers and enablers were grouped into two deductively developed themes, each addressing one of the target behaviours.

Each theme was classified into subthemes based on the COM‐B components [20]. The themes and subthemes were summarised narratively. In addition, behavioural analysis tables were created using a similar approach to intervention development studies [35, 36]. This provided a transparent way of mapping the barriers and enablers to the COM‐B components, TDF domains, and original intervention features or adaptations. The behavioural analysis tables were discussed and refined by the project team. Further feedback on the analysis process and findings was obtained through PAG meetings.

### 2.7 Phase 1b: Developing and Finalising the Adapted Intervention

#### 2.7.1 Overview

Phase 1b involved developing and finalising the adapted OA peer‐mentorship intervention. Provisional adaptations were undertaken based on the focus group/interview findings and project team members' suggestions. The team members' suggestions focused on addressing considerations identified in the previous study of the in‐person OA peer mentorship intervention [14]; updating the content to align with the current National Institute for Health and Care Excellence (NICE) OA guideline [37]; and ensuring the intervention can be delivered remotely in an inclusive and acceptable way. The participatory workshops and mentorship delivery practice runs described below were then undertaken to guide further adaptations and finalise the intervention.

#### 2.7.2 Participatory Workshops

Two participatory workshops, each lasting 2 h, were held in November 2023. Both were conducted online via Microsoft Teams due to the geographical dispersion of attendees. To ensure diverse perspectives were considered, attendees included:

- Five people with hip/knee OA experiencing socioeconomic disadvantage recruited by sharing study advertisements/information via community organisations, a university public engagement group, and a previous community engagement project.
- Four community organisation representatives recruited by approaching six Leeds‐based community organisations that work with people with hip/knee OA experiencing socioeconomic disadvantage.
- Six PPI representatives, two of whom were peer mentors in the previous study, recruited through the study PPI group.

The attendees were diverse in their ethnicity, gender, confidence in using the Internet, experience of OA, and the region of England they were based in. Public attendees were offered support with Microsoft Teams, a £30 recognition payment, and reimbursement for WiFi costs.

Before the workshops, the attendees were given a document summarising the study background, workshop purpose, and workshop plans. Each attendee was invited to attend one workshop only. Both workshops were co‐facilitated by three researchers (A.M.A., E.L., A.D.O.) following an agenda (Table 2).

**Table 2:** Participatory workshop agenda.

Activity	Overview^a^
Welcome and group introductions	The facilitators welcomed all attendees, and everyone introduced themselves.
Introduction to the workshop	A facilitator briefly introduced the plan for the workshop and clarified key terms such as ‘peer mentor’ and ‘mentee’.
Peer mentorship intervention content	A facilitator shared drafts of the peer mentor resource pack and participant handouts on screen. Attendees were encouraged to discuss their views of the resources pack, handouts, and overall intervention content. The core topics ‘Eating well, feeling well’ and ‘Building an active lifestyle’ were discussed in depth as earlier work suggested those topics would be particularly sensitive/challenging to cover.
Break	All attendees took a brief break.
Peer mentor training	A facilitator outlined the provisional plan for training the peer mentors. Attendees were encouraged to discuss their views of the training content and delivery.
Peer mentorship intervention format	A facilitator outlined potential options for the peer mentorship intervention format: six sessions delivered one‐to‐one; six sessions delivered by one peer mentor to a small group of mentees; or two sessions delivered one‐to‐one followed by four sessions delivered by one peer mentor to a small group of mentees. Attendees were encouraged to discuss their views of each format.
Thank you and close	The facilitator thanked all attendees.

^a^ The activities were largely undertaken in the order listed, but a flexible approach was employed, and some discussions related to the activities overlapped.

PowerPoint slides were shared onscreen to support the workshop activities. With the attendees' consent, the workshops were recorded and automatically transcribed using the built‐in functionality of Microsoft Teams. Following each workshop, the attendees were asked to provide feedback via a brief online questionnaire.

#### 2.7.3 Mentorship Delivery Practice Runs

Four members of the wider PPI group each took part in two practice runs of delivering peer mentorship sessions. The PPI members provided feedback by completing a form with questions focusing on the delivery and accessibility of the resources, connecting and communicating online, content of the resource pack and handouts, working through the ‘Getting active, staying active’ topic and any other feedback.

#### 2.7.4 Intervention Description

The suggested adaptations from the participatory workshops and mentorship delivery practice runs were documented in tables along with whether each adaptation was made and the rationale for making/not making the adaptation. The final decisions about whether to make adaptations were agreed upon through team discussions. All the adaptations made were coded using the FRAME [21] by one researcher (A.M.A.) and verified by a second researcher (M.C.). A process‐oriented logic model of the adapted intervention was created by revising the original intervention logic model [14].

## 3 Results

### 3.1 Phase 1a: Exploring Barriers and Enablers

#### 3.1.1 Participants

**Figure 2:**
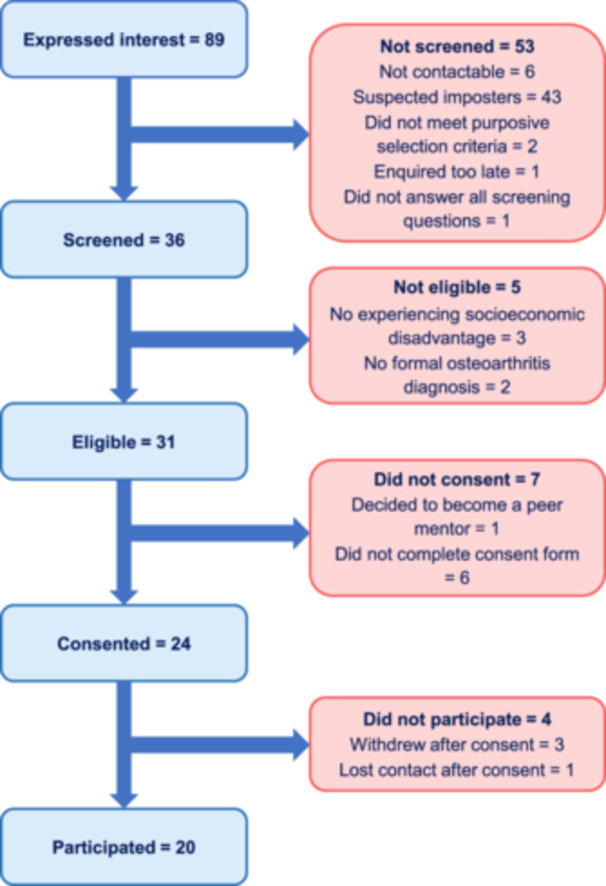
Flow chart for participants in the Phase 1a qualitative descriptive study. An image description of the flow chart is available in Supporting File S1.

Eighty‐nine individuals contacted the research team to express an interest in participating, of whom 36 were screened, 31 were eligible, 24 consented and 20 participated. Figure 2 provides the reasons for exclusion at each stage.

Table 3 presents the participant characteristics. The participants are referred to below by their pseudonym, gender and data collection format.

**Table 3:** Phase 1 participant characteristics.

Characteristic	Number (%)
Gender	
Male	8 (40)
Female	12 (60)
Age (years)	
30–39	1 (5)
40–49	1 (5)
50–59	5 (25)
60–69	7 (35)
70–79	5 (25)
80–89	1 (5)
Ethnicity	
Black British	3 (15)
British East European	1 (5)
British Indian	4 (20)
British Pakistani	4 (20)
East African Asian	2 (10)
English American	1 (5)
White British	5 (25)
Site of osteoarthritis	
Knee	13 (65)
Hip	2 (10)
Both	5 (25)
Time living with OA (years)	
2 < 5	4 (20)
5 < 10	8 (40)
10 < 20	3 (15)
≥ 20	5 (25)
Consider themselves to have a disability	
Yes – because of OA	15 (75)
Yes – because of OA plus other conditions	2 (10)
Yes – because of a condition(s) other than OA	1 (5)
No	2 (10)
Have access to the Internet	
Yes	18 (90)
No	2 (10)
Experience of using videoconferencing	
Not at all experienced	6 (30)
Fairly experienced	1 (5)
Quite experienced	4 (20)
Experienced	4 (20)
Very experienced	5 (25)

Abbreviation: OA, osteoarthritis.

#### 3.1.2 Overview

Numerous barriers and enablers to the target behaviours were identified and grouped into two deductively developed themes:

- Theme 1: Barriers and enablers to using self‐management strategies
- Theme 2: Barriers and enablers to attending remote OA peer mentorship sessions

Details and illustrative quotes about the barriers and enablers are provided for each theme below, classified into subthemes based on the COM‐B components.

#### 3.1.3 Theme 1: Barriers and Enablers to Using Self‐Management Strategies

The Theme 1 barriers and enablers span all the COM‐B components except for physical capability and eight TDF domains. Most barriers and enablers were addressed by the original intervention features, so only a few adaptations were required (Table 4).

**Table 4:** Behavioural analysis for using self‐management strategies.

COM‐B component	TDF domain	Barriers [B] and enablers [E]	Original intervention features [F] and adaptations [A]
Psychological capability	Knowledge	Insufficient knowledge about OA and self‐management strategies [B] Knowledge about OA and self‐management strategies [E]	Include ‘Self‐managing osteoarthritis’ as a core topic, with facts and myths about OA [F] Include core topics on key self‐management strategies, including physical activity/exercise, weight management, and activity pacing [F]
	Skills	Difficulty finding or understanding relevant self‐management information online [B] Being able to find relevant self‐management information online [E]	Provide self‐management support, including a resource pack [F] Adapt the wording and formatting of the resource pack and mentee handouts to improve readability, clarity, and accessibility [A] Add extra links to national support organisations to the ‘Getting connected’ topic in the participant handouts [A]
Physical opportunity	Environmental context and resources	Use of aids or resources (including cushioned trainers) to self‐manage [E] Access to sports/health facilities [E] Lack of infrastructure e.g. transport [B]	Include the Versus Arthritis booklet ‘Footcare and footwear’ as an extra document with the handouts if indicated [F] Provide tailored support, accounting for individual needs and circumstances [F]
Social opportunity	Social influences	Limitations with support from friends, family, and groups [B] Support from friends, family, and groups [E]	Change ‘Getting connected’ to an optional topic [A] Ensure all peer mentors have lived experience of OA and are trained [F]
		Inadequate support from health and care professionals [B] Support from health and care professionals [E]	Ensure all peer mentors have lived experience of OA and are trained [F]
Reflective motivation	Beliefs about capabilities	Mobility/physical limitations due to OA [B] Mobility/physical limitations due to other health conditions [B]	Provide tailored support, including a tailored home exercise programme [F]
	Beliefs about consequences	Believing physical activity/exercise will cause pain [B] Believing physical activity/exercise is beneficial [E]	Explain the benefits of exercise and address negative beliefs [F]
		Believing that pain management medications are ineffective [B]	Change ‘Managing pain’ to a core topic, with guidance about nonmedical forms of pain management and guidance/signposting related to medications [A]
	Intentions	Feelings of helplessness, depression, or anxiety leading to demotivation [B]	Include signposting to professional mental health support in the ‘Getting motivated’ handout [F]
		Personal motivations for self‐management [E]	Provide tailored support, including support with setting personal goals [F]
Automatic motivation	Emotions	Fear of going out [B]	Provide tailored support, including addressing individual fears [F]

Abbreviations: COM‐B, Capability, Opportunity, Motivation Model of Behaviour (1); OA, osteoarthritis; TDF, Theoretical Domains Framework (2).

##### 3.1.3.1 Psychological Capability

Insufficient knowledge about OA and its management appeared to present a barrier to using self‐management strategies for a few individuals. For example, when one individual was asked how she felt a peer mentor talking to her about OA and its management might benefit her, she responded:

To know more about it really because I don't know much about it.

(Audrey, female, telephone interview)

While a couple of focus group participants appeared able to find relevant self‐management information online, others reported difficulty finding online information they could trust and understand. Despite these barriers, many participants demonstrated knowledge of specific self‐management strategies, such as healthy eating and physical activity.

##### 3.1.3.2 Physical Opportunity

Numerous focus group participants and interviewees highlighted aids or resources they find helpful, such as walking aids, cushioned trainers, home adaptations, heat, and massage. Having access to a swimming pool, hydrotherapy, or gym appeared to be an enabler to physical activity for a small number of participants. Conversely, one interviewee highlighted lack of infrastructure as an issue:

We've no buses, they've cut off our Sunday bus completely, we've no shops, we've no infrastructure down here that would help people make life easier.

(Nicola, female, telephone interview)

##### 3.1.3.3 Social Opportunity

Support from family, friends, and groups was highlighted as particularly helpful by some participants. Valued support included advice from trusted friends or peers, practical support, emotional support, and having other people to do activities with:

And at that point she says, mum, will you do it if I do it with you, you have to follow this way, you have to eat this and that? And I was in tears and she could see that and then just my other daughter rang at that point as well and she says, I'll join you as well, mum, we'll all do it together.

(Kamala, female, in‐person focus group)

A few participants highlighted limitations with support from family, friends, and groups, for example due to lack of availability and not having personal experience of OA. Several reported inadequate support from health and care professionals due to issues such as the professionals appearing disinterested, being too focused on prescribing medications, or not providing personalised support. Conversely, some participants reported positive experiences with professionals such as doctors, social prescribers, or physiotherapists.

##### 3.1.3.4 Reflective Motivation

Mobility/physical limitations due to OA were mentioned during all the focus groups and interviews. Some participants reported their pain as excruciating and highlighted they require adjustments to complete daily tasks. Many reported that their activities are restricted. Balancing rest and activity was highlighted as a specific challenge. Multiple participants also reported other health conditions that limit their mobility/physical abilities, with a few believing their different conditions may compound each other:

[…] if I didn't have the osteoarthritis, maybe I would be able to deal with the other conditions a bit better.

(Fiaz, female, telephone interview)

Some participants were concerned that physical activity/exercise would aggravate their pain, for example due to doing exercises incorrectly. In contrast, multiple other participants highlighted physical activity/exercise as helpful, with a few reporting how they had benefited from exercising themselves. Another belief expressed by a few participants was that pain management medications are ineffective. Feelings of helplessness, depression, or anxiety due to the pain of their OA and/or restrictions in their activities were reported by a few participants, and could contribute to participants feeling demotivated:

But after, but during covid it's got worse and worse and therefore I can't get to some of those things that I used to do which is very frustrating and I've just got to try and restrict myself to what I can do and that's what I find and it can be demotivating in many ways and upsetting.

(Sheila, female, online focus group)

Personal motivations for self‐management, such as wanting to spend time with their children or avoid having surgery, were highlighted as important by a few participants.

##### 3.1.3.5 Automatic Motivation

One interviewee and a focus group participant reported being afraid to go out due to the risk of falling/their knees buckling or being a target of hate crime:

The other thing as well is obviously in terms of using sticks and whatever there, there's a stigma against disabled people and also there's a fear factor that people will pick on somebody who uses a stick et cetera, you know, to attack you or do things.

(Shafaq, male, online focus group)

#### 3.1.4 Theme 2: Barriers and Enablers to Attending Remote OA Peer Mentorship Sessions

The Theme 2 barriers and enablers span all the COM‐B components except for physical capability and seven TDF domains. While a few Theme 2 barriers and enablers were addressed by the original intervention features, most required adaptations (Table 5).

**Table 5:** Behavioural analysis for attending remote osteoarthritis peer mentorship sessions.

COM‐B component	TDF domain	Barriers [B] and enablers [E]	Original intervention features [F] and adaptations [A]
Psychological capability	Knowledge	Not knowing what peer mentorship is [B] Knowing what peer mentorship is [E]	Provide a tailored explanation of peer mentorship as part of the recruitment to the intervention and during the matching process [F] Adapt the wording of the mentee recruitment materials to clarify the concept of peer mentorship [A]
	Skills	Experience of making video calls with a specific videoconferencing platform [E]	Offer flexibility with the choice of videoconferencing platform [A]
Physical opportunity	Environmental context and resources	Lack of access to a digital device and adequate internet connection [B]	Offer mentees a loan digital device and Wi‐Fi support funds [A]
		Difficulty engaging at specific times of day [B]	Offer flexibility with the timing of the mentorship sessions and consider timing preferences during matching [F]
		Lack of time to engage in peer mentorship [B]	Cannot be directly addressed.
Social opportunity	Social influences	Female peer mentor or all‐female group [E]	Offer the option of same‐gender matching [F]
		Peer mentor who speaks the same language or an interpreter present [E]	Offer the option of having a peer mentor who speaks the same language or an interpreter present [A]
		Peer mentor with lived experience of OA [E]	Ensure all peer mentors have lived experience of OA [F]
		Empathetic peer mentor [E]	Include guidance on being empathetic in the peer mentor training [F]
Reflective motivation	Beliefs about capabilities	Low confidence in ability to make videoconferencing calls [B]	Offer the option to hold mentorship sessions via telephone [A] Offer mentees reassurance about the remote format and digital coaching/support [A]
	Beliefs about consequences	Concerns about peer mentorship [B] Believing peer mentorship would be beneficial [E]	Offer tailored peer mentorship which focuses on the mentee's desired benefits and addresses their concerns [F]
		Concerns about a remote format [B] Believing a remote format would be appropriate or advantageous [E]	Offer the option to hold mentorship sessions via telephone or videoconferencing [A] Offer mentees reassurance about the remote format and digital coaching/support [A]
		Believing a group format would be advantageous [B] Believing a one‐to‐one format would be advantageous [E]	Offer the option of having mentorship in small groups (rather than one‐to‐one only) if feasible [A]
Automatic motivation	Emotion	Feeling fed up with or stressed by videoconferencing calls [B]	Offer the option to hold mentorship sessions via telephone or videoconferencing [A] Offer mentees reassurance about the remote format and digital coaching/support [A]

Abbreviations: COM‐B, Capability, Opportunity, Motivation Model of Behaviour (1); OA, osteoarthritis; TDF, Theoretical Domains Framework (2).

##### 3.1.4.1 Psychological Capability

A couple of interviewees stated they did not know what peer mentorship is. While a few focus group participants reported some understanding of peer mentorship, their views ranged from ‘someone telling you what you should, you could be doing to help yourself’ to a more nuanced understanding of a peer mentor as someone with OA who ‘can pass information and their experiences on’ and provide mutual support/encouragement.

An important facilitator related to engaging with remote support appeared to be prior experience of making videoconferencing calls:

Yeah, because I'm used to it now. All the Zoom meetings and everything, I can do it.

(Arin, female, in‐person focus group)

Correspondingly, a couple of interviewees highlighted they would want the peer mentorship to be provided using a videoconferencing platform they were familiar with.

##### 3.1.4.2 Physical Opportunity

A few focus group participants highlighted lack of access to a digital device and/or adequate internet connection as a barrier to making videoconferencing calls. One linked this to financial concerns:

And unfortunately, in this day and age where people are struggling with money and things that are going to be cut back, and it could be Internet and things like that that are cut back, I like to say.

(Angela, female, online focus group)

One focus group participant reported he would have problems attending sessions too early or late in the day due to difficulty getting up and falling asleep respectively, while an interviewee reported she does not have time to engage in activities for herself due to supporting a family member with dementia.

##### 3.1.4.3 Social Opportunity

The importance of having a peer mentor with lived experience of OA was evident across most focus groups and interviews. However, there were differing opinions in one focus group about whether a mentor needed to have more experience/knowledge of OA than their mentee:

The argument to use someone experienced, you know it makes sense, but also the opposite argument that if you have somebody who's not so experienced, so more the peer to peer thing, somebody who's similar is starting on, on a journey where they've just been diagnosed with osteoarthritis, that may also perhaps work because you [are] kind of discovering new things, new treatments together.

(Aadil, male, online focus group)

Having an empathetic peer mentor appeared to be a key facilitator to attending mentorship sessions. Four interviewees highlighted the importance of having a female peer mentor or all‐female group for personal or cultural reasons. Additionally, the interviewee with a Patwari interpreter stated she would need a peer mentor who spoke her language or interpreter present.

##### 3.1.4.4 Reflective Motivation

Low confidence in their ability to make videoconferencing calls appeared to be a barrier for multiple participants. Some felt receiving digital support would help address that, whereas others felt they would struggle to learn/remember how to make videoconferencing calls.

Not on the tablet. I've got learning difficulties, so, like I said, I don't know if I'd pick it [up] and that.

(Audrey, female, telephone interview)

Concerns about peer mentorship were raised in a few focus groups and interviews, including whether the peer mentorship could account for people's individual needs and the risk of the peer mentor becoming controlling. Conversely, numerous potential benefits of OA peer mentorship were identified across all the focus groups and interviews. These related to the peer mentor sharing experiences and providing personalised holistic support, including emotional support, encouragement, motivation, information, and practical advice. Having space to share and feel listened to was also considered important:

[…] it'd be very beneficial because it's still being able to speak to somebody and discuss all the things that…you know, the daily things that affect you with this pain and how to manage, but also somebody's there listening to you as well.

(Rifat, female, online interview)

Participants' perspectives about delivering peer mentorship remotely varied widely. Key concerns were that remote interactions are ‘stilted’, ‘cold’ and ‘too pedantic’, and people may learn less due to factors such as missing out on body language. While some participants reported liking videoconferencing calls due to being able to see people, one interviewee reported she would only be happy to receive remote support via telephone. Despite the concerns raised, most participants felt a remote format would be appropriate or even advantageous. Suggested advantages included the possibility of making a recording to play back later, practical benefits such as not needing to travel, and time and money savings:

You don't have the travelling time and the waiting time, so my day's short anyway because of lack of sleep. So taking those factors out of the day makes life a lot easier.

(Michael, male, online focus group)

Participants' preferences for a one‐to‐one or group format also varied. Some liked the idea of being able to learn from other people's experiences in a group. Others stated they would prefer one‐to‐one support because it would be more personalised, and some people may not fit in or have confidence to share in a group. Offering people choices of online, remote and hybrid formats, and one‐to‐one or group options, was suggested as a potential strategy for meeting people's individual needs.

##### 3.1.4.5 Automatic Motivation

Participants from the in‐person focus group expressed negative emotions about videoconferencing calls, reporting being ‘really fed up’ and ‘angry’ about the shift towards everything being online. An interviewee also reported she does not make videoconferencing calls due to finding them stressful:

I'm not good with it [making video calls] and it stresses me, so anything that stresses me, I don't do […]

(Nicola, female, telephone interview)

### 3.2 Phase 1b: Developing and Finalising the Adapted Intervention

Tables 6 and 7 summarise all the adaptations made to the peer mentorship programme and peer mentor training, respectively, coded using the FRAME [21]. Most adaptations were made based on the focus group/interview findings, project team members' suggestions, and participatory workshops. Feedback from the mentorship delivery practice runs reinforced the need for a few adaptations and led to five additional adaptations. A small number of points raised during the participatory workshops and practice runs were not addressed. Supporting File S4 summarises these points and provides further details about the rationale for each adaptation.

**Table 6:** Summary of adaptations to the peer mentorship programme.

Area	Adaptation^a^	Driver(s)^b^	What	Level of delivery^c^	Goal(s)	Reason(s)^d^
Peer mentorship programme delivery	Offer six support sessions	PT	Content: shortening/condensing (pacing/timing)	Target intervention group	Increase retention Reduce cost	Organisation/setting: available resources Recipient: motivation and readiness
	Offer the option to hold mentorship sessions via telephone or videoconferencing	FG/I PT	Contextual: format	Individual	Increase reach or engagement Reduce cost	Organisation/setting: available resources Recipient: motivation and readiness
	Offer flexibility with the choice of videoconferencing platform	FG/I PW MDP	Contextual: format	Individual	Increase reach or engagement	Recipient: literacy and education level
	Ensure the peer mentors who deliver the programme consider themselves to be experiencing socioeconomic disadvantage	PT	Contextual: personnel	Target intervention group	Increase reach or engagement	Recipient: motivation and readiness
	Offer the option of having a peer mentor who speaks the same language or an interpreter present	FG/I PT PW	Contextual: personnel	Individual	Increase reach or engagement	Recipient: first/spoken languages
	Offer the option of having mentorship in small groups if feasible	FG/I PW	Contextual: format	Individual	Increase reach or engagement	Recipient: motivation and readiness
	Offer mentees reassurance about the remote format and digital coaching/support	FG/I PT PW MDP	Contextual: personnel	Individual	Increase reach or engagement	Recipient: motivation and readiness
	Offer support for mentees with additional communication needs (e.g. for people who are Deaf or hard of hearing)	PT PW	Contextual: personnel	Individual	Increase reach or engagement	Recipient: first/spoken languages; comorbidity/multimorbidity
	Provide mentees with printed and electronic copies of the mentee handouts before their first support session	PT PW MDP	Contextual: format	Target intervention group	Increase reach or engagement Improve feasibility	Recipient: access to resources
Peer mentorship programme content and resources	Adapt the wording of the mentee recruitment materials to clarify the concept of peer mentorship	FG/I	Content: tailoring/tweaking/refining	Target intervention group	Improve fit with recipients	Recipient: literacy and education level
	Split the peer mentor resource pack and mentee handouts into two separate documents with the acknowledgements at the end of each document	PT	Content: tailoring/tweaking/refining	Target intervention group	Improve fit with recipients	Recipient: literacy and education level
	Change the images to maximise diversity and relevance for peer mentors and participants	PT	Content: tailoring/tweaking/refining	Target intervention group	Increase reach or engagement Address cultural factors	Recipient: race; ethnicity; motivation and readiness
	Add guidance to the resource pack on providing support remotely and amend the document's structure accordingly	PT	Content: adding elements	Target intervention group	Improve feasibility	Recipient: motivation and readiness
	Adapt the wording and formatting of the resource pack and mentee handouts to improve readability, clarity, and accessibility	FG/I PT PW	Content: tailoring/tweaking/refining	Target intervention group	Improve fit with recipients	Recipient: literacy and education level; physical capacity
	Adapt the content of the resource pack and handouts to ensure it aligns with the current NICE OA guideline (3) and relevant evidence	PT	Content: tailoring/tweaking/refining	Target intervention group	Improve effectiveness/outcomes	Sociopolitical: existing policies
	Change ‘Managing pain’ to a core topic	FG/I PT	Content: tailoring/tweaking/refining	Target intervention group	Improve effectiveness/outcomes	Recipient: motivation and readiness
	Change ‘Eating well, feeling well’ to a core topic	PT	Content: tailoring/tweaking/refining	Target intervention group	Improve effectiveness/outcomes	Recipient: motivation and readiness
	Change ‘Getting connected’ to an optional topic	FG/I PT	Content: tailoring/tweaking/refining	Target intervention group	Improve effectiveness/outcomes	Recipient: motivation and readiness
	Combine the ‘Building muscle strength’ core topic and ‘Building an active lifestyle’ optional topic into a single ‘Getting active, staying active’ core topic	PT	Content: tailoring/tweaking/refining	Target intervention group	Improve effectiveness/outcomes	Recipient: motivation and readiness
	Combine the ‘Breathing and relaxation’ and ‘Sleep quality’ optional topics into one ‘Sleeping and resting’ optional topic	PT	Content: tailoring/tweaking/refining	Target intervention group	Improve effectiveness/outcomes	Recipient: motivation and readiness
	Add ‘Having a joint replaced’ as an optional topic	PT PW	Content: adding elements	Target intervention group	Improve effectiveness/outcomes	Recipient: motivation and readiness
	Add ‘Getting support for work’ as an optional topic	PT	Content: adding elements	Target intervention group	Improve effectiveness/outcomes	Recipient: motivation and readiness
	Adapt the ‘Setting realistic goals’ topic in the resource pack to provide clearer guidance on how to set action plans and include different goal examples	PT	Content: tailoring/tweaking/refining	Target intervention group	Improve effectiveness/outcomes	Recipient: motivation and readiness
	Add guidance on weight management to the ‘Eating well, feeling well’ topic in the resource pack and mentee handouts, including guidance for peer mentors on how to approach the topic sensitively	PT PW	Content: adding elements	Target intervention group	Address cultural factors Improve effectiveness/outcomes	Recipient: cultural or religious norms; motivation and readiness
	Offer culturally adapted versions of the ‘Eatwell guide’ in the mentee handouts	PW	Content: adding elements	Individual	Address cultural factors	Recipient: cultural or religious norms
	Adapt the ‘Getting active, staying active’ topic text in the resource pack to emphasise that physical activity is usually helpful not harmful	PW	Content: tailoring/tweaking/refining	Target intervention group	Improve effectiveness/outcomes	Recipient: motivation and readiness
	Adapt the text in the ‘Getting active, staying active’ topic in the mentee handouts to highlight that the activity plan can be undertaken in any comfortable clothing	PW	Content: tailoring/tweaking/refining	Target intervention group	Improve fit with recipients	Recipient: motivation and readiness
	Provide the Versus Arthritis ‘Let's Move Tracker’ as an extra document in the ‘Getting active, staying active’ topic	PT	Content: adding elements	Target intervention group	Improve effectiveness/outcomes	Recipient: motivation and readiness
	Add a safety checklist to the ‘Getting active, staying active’ topic in the mentee handouts	MDP	Content: adding elements	Target intervention group	Improve feasibility	Recipient: physical capacity
	Include the Versus Arthritis booklet ‘Footcare and footwear’ as an extra document with the handouts if indicated	FG/I MDP	Content: adding elements	Individual	Improve effectiveness/outcomes	Recipient: motivation and readiness
	Include the Versus Arthritis booklet ‘Sleep and arthritis’ as an extra document with the handouts if indicated	MDP	Content: adding elements	Individual	Improve effectiveness/outcomes	Recipient: motivation and readiness
	Include the Versus Arthritis ‘Eating well with arthritis’ booklet as an extra document with the mentee handouts for all mentees	MDP	Content: adding elements	Target intervention group	Improve effectiveness/outcomes	Recipient: motivation and readiness
	Add a reminder to regularly recap on previously covered topics to the ‘Delivering support sessions’ section of the resource pack	PW	Content: adding elements	Target intervention group	Improve effectiveness/outcomes	Recipient: motivation and readiness
	Remove the links to local support organisations from the ‘Getting connected’ topic in the mentee handouts	PT	Content: removing/skipping elements	Target intervention group	Improve feasibility	Organisation/setting: location/accessibility
	Add extra links to national support organisations to the ‘Getting connected’ topic in the mentee handouts, including links for local group search functions, and highlight that mentees could search for other opportunities	FG/I PT PW	Content: adding elements	Target intervention group	Improve feasibility	Recipient: access to resources
	Add extra pages for mentees to make notes at the end of the mentee handouts	PT MDP	Content: adding elements	Target intervention group	Increase reach or engagement	Recipient: access to resources
	Offer mentees a loan digital device and WiFi support funds	FG/I PT MDP	Content: adding elements	Individual	Increase reach or engagement	Recipient: access to resources

Abbreviations: FG/I, focus groups or interviews; MDP, mentorship delivery practice; NICE, National Institute for Health and Care Excellence; OA, osteoarthritis; PW, participatory workshops; PT, project team.

^a^ All the adaptations were planned/proactive adaptations undertaken before feasibility testing of the adapted intervention and were intended to be fidelity consistent.

^b^ ‘Driver(s)’ refers to the main activity/activities that led to the adaption. Details of who was involved in each activity are provided in the main text.

^c^ ‘Target intervention group’ refers to adults with hip/knee osteoarthritis who are experiencing socioeconomic disadvantage.

^d^ ‘Recipient’ refers to the mentee in this table.

**Table 7:** Summary of adaptations to the peer mentor training.

Area	Adaptation^a^	Driver^b^	What	Level of delivery^c^	Goal(s)	Reason^d^
Peer mentor training programme delivery	Deliver the training remotely via videoconferencing	PT	Training and evaluation: format	Target intervention group	Increase reach or engagement Reduce cost	Organisation/setting: available resources
	Offer peer mentors digital coaching/support	PT PW MDP	Training and evaluation: personnel	Individual	Increase reach or engagement	Recipient: motivation and readiness
	Deliver the training over two nonconsecutive days	PT PW	Training and evaluation: lengthening/extending (pacing/timing)	Target intervention group	Increase reach or engagement	Recipient: motivation and readiness
	Incorporate more breaks into the training	PW	Training and evaluation: lengthening/extending (pacing/timing)	Target intervention group	Increase reach or engagement	Recipient: motivation and readiness
	Use breakout rooms to provide opportunities for small group discussions and role play	PW	Training and evaluation: format	Target intervention group	Increase reach or engagement	Recipient: motivation and readiness
	Include peer mentors from the previous study as facilitators for role play and discussion	PT PW	Training and evaluation: personnel	Target intervention group	Increase reach or engagement	Recipient: motivation and readiness
	Provide peer mentors with printed and electronic copies of the resource pack and mentee handouts, and the administrative documentation (e.g., confidentiality form), before the training	PT PW MDP	Training and evaluation: format	Target intervention group	Increase reach or engagement Improve feasibility	Recipient: access to resources
	Provide peer mentors with brief verbal information about their mentees' self‐management needs before the introductory session (with the mentees' consent)	PT PW MDP	Training and evaluation: adding elements	Target intervention group	Improve effectiveness/outcomes	Recipient: motivation and readiness
Peer mentor training programme content	Include training on how to provide remote support with the home exercise plan and tailor the exercise plan for mentees who have restricted mobility and/or use mobility aids	PW	Training and evaluation: adding elements	Target intervention group	Improve effectiveness/outcomes	Recipient: motivation and readiness
	Include additional emphasis that physical activity is usually helpful not harmful	PW	Training and evaluation: adding elements	Target intervention group	Improve effectiveness/outcomes	Recipient: motivation and readiness
	Include a suggestion about putting a smartphone (if used) on speaker during the mentorship sessions	MDP	Training and evaluation: adding elements	Target intervention group	Improve feasibility	Recipient: motivation and readiness
	Include information about considering falls risk when recommending outdoor activities	MDP	Training and evaluation: adding elements	Target intervention group	Improve effectiveness/outcomes	Recipient: motivation and readiness
	Encourage peer mentors to ensure they regularly recap on previously covered topics during the mentorship sessions	PW	Training and evaluation: adding elements	Target intervention group	Improve effectiveness/outcomes	Recipient: motivation and readiness
	Offer peer mentors a loan digital device and WiFi support funds	PT MDP	Training and evaluation: adding elements	Individual	Increase reach or engagement	Recipient: access to resources

Abbreviations: FG/I, focus groups or interviews; MDP, mentorship delivery practice; PW, participatory workshops; PT, project team.

^a^ All the adaptations were planned/proactive adaptations undertaken before feasibility testing of the adapted intervention and were intended to be fidelity consistent.

^b^ ‘Driver(s)’ refers to the main activity/activities that led to the adaption. Details of who was involved in each activity are provided in the main text.

^c^ ‘Target intervention group’ refers to adults with hip/knee osteoarthritis who are experiencing socioeconomic disadvantage.

^d^ ‘Recipient’ refers to the peer mentor in this table.

Feedback on the workshops was provided by 67% of attendees. Workshops were positively evaluated as being interesting, informative and well‐organised. Attendees appreciated having materials sent to them beforehand and felt encouraged to contribute and raise any concerns about the proposed intervention.

**Figure 3:**
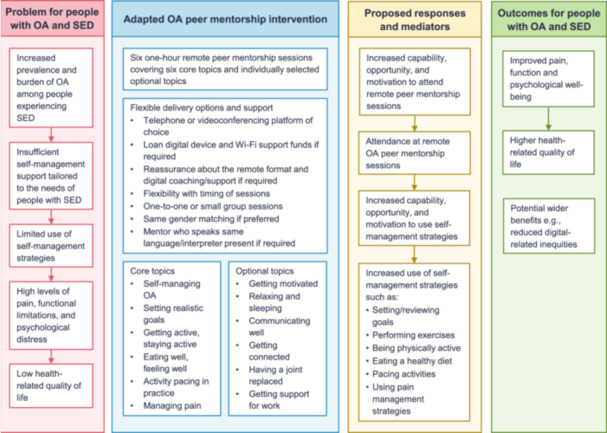
Logic model of the adapted osteoarthritis peer mentorship intervention. This figure is a modified version of Figure 1 in Anderson et al. [14] created under the terms of the Creative Commons Attribution 4.0 Unported (CC BY 4.0) license (https://creativecommons.org/licenses/by/4.0/). An image description of the logic model is available in Supporting File S1. OA, osteoarthritis; SED, socioeconomic disadvantage.

The finalised adapted OA peer mentorship intervention involves six 1‐h self‐management support sessions delivered remotely by a trained peer mentor. Mentees can express a preference to receive the support sessions individually or in a small group via telephone or a videoconferencing platform of their choice. Figure 3 provides a logic model of the intervention. Supporting File S5 provides examples of the implementation of the key behaviour change techniques (BCTs). As with the previous intervention, the implementation of BCTs and the choice/order of topics is flexible and participant led, but peer mentors are encouraged to cover all the core topics at least once and set/review goals with the mentee each week.

## 4 Discussion

This study's multi‐method approach enabled an in‐person OA peer mentorship intervention to be rigorously and systematically adapted for remote delivery to people identifying as experiencing socioeconomic disadvantage. A wide range of adaptations were made, spanning the content and delivery of the peer mentor training, peer mentorship sessions, and supporting resources. Coding the adaptions using the FRAME [21] demonstrated substantial diversity in the types of adaptations, their goals, and the reasons they were made. Numerous adaptations were made at the target group level, and so would apply whenever the intervention is delivered. A smaller number of adaptations were made at the individual level. These would only apply to individuals with particular characteristics, such as lack of access to a digital device.

The barriers and enablers to using self‐management strategies identified in this study largely align with those reported in previous literature. For example, this study and previous studies have highlighted that unhelpful beliefs about OA can present barriers to self‐management [11, 16, 38]. This has led to a global call to action from the ‘Change OA Narrative’ (COAN) Initiative [11]. The suggested new narrative frames OA within the biopsychosocial framework and promotes a strengths‐based approach aimed at empowering people to make healthy lifestyle changes and use self‐management strategies. The COAN Initiative emphasise the new narrative needs to be shared in acceptable, accessible, and easily understandable ways [11]. Correspondingly, this study adapted the wording and formatting of the resource pack and mentee handouts to improve readability, clarity, and accessibility.

As highlighted in Tables 4 and 5, many of the barriers and enablers identified in this study did not require adaptations as they were already addressed by the in‐person OA peer mentorship intervention. For example, the facilitator ‘Personal motivations for self‐management’ was already addressed through the provision of tailored support, including support with setting personal goals. This could potentially help increase mentees' intrinsic motivation for using self‐management strategies.

Another action recommended by the COAN Initiative is to develop culturally relevant self‐management programmes [11]. This study provides examples of cultural adaptations, such as offering culturally adapted versions of the ‘Eatwell guide’ [39, 40, 41]. The importance of considering culture was also highlighted by Woodward et al. [10] in a qualitative study of self‐management of multiple long‐term conditions among people experiencing socioeconomic deprivation. As in this study, Woodward et al. [10] reported different conditions may exacerbate each other, highlighting the need for personalised and holistic self‐management support. This study's participants largely felt that peer mentors could play a valuable role in providing such support. This corresponds with qualitative findings from the feasibility study of the in‐person OA peer mentorship intervention, in which **‘**Making the intervention individually relevant’ was identified as an enabler of self‐management [16].

Many of the other barriers and facilitators identified by Woodward et al. [10] overlap with this study's findings. For example, Woodward et al. [10] highlighted potential benefits and limitations of informal social support. They also reported that technology can play empowering and disempowering roles in supporting self‐management and suggested that policymakers should invest in supporting populations experiencing socioeconomic disadvantage to access digital health resources. This study's adapted intervention aims to support digital inclusion by offering a loan digital device, WiFi support funds, reassurance about the remote format, digital coaching/support, and a choice of videoconferencing platforms. Importantly, the adapted intervention can also be provided to people who are unwilling or unable to use digital technologies through the provision of printed resources and telephone support. This addresses recommendations from groups such as ARMA and NHS England, which emphasise the importance of offering non‐digital support options [8, 42]. This flexible approach is also likely to support possible roll out and sustainability of the intervention by addressing barriers related to digital awareness, literacy and access.

### 4.1 Strengths and Limitations

Key strengths of this study include its rigorous multi‐method approach, inclusion of diverse partners, and extensive PPI. The participants varied in key characteristics such as their ethnicity and experience of using videoconferencing, which helped ensure differing perspectives were considered. While many adaptations were made based on the focus group/interview findings and project team members' suggestions, a range of additional adaptations were identified through the participatory workshops and mentorship delivery practice runs (Tables 6 and 7). This demonstrates the added value of employing a combination of activities with different individuals. Feedback about the PPI activities suggested they went well, with PPI representatives feeling their comments were actively considered and taken on board. A key benefit of the PPI was the generous sharing of information regarding self‐management of OA from people with lived experience, which could then be passed onto the peer mentors and their mentees. In addition, a few PPI representatives acted as ambassadors for the study within their communities. This enabled cultural sensitivity and local recruitment to take place through a ‘trusted source’.

Creating behavioural analysis tables based on the COM‐B model [20] and TDF [18, 19] and coding the adaptations using the FRAME [21] provided a theoretically informed and transparent way of presenting this study's findings. These processes also have limitations. For example, the behavioural analysis tables do not account for overlap between TDF domains, and some of the adaptations did not fit well with the ‘goals’ and ‘reasons’ specified in the FRAME. Involving multiple authors in the mapping/coding processes helped ensure a range of perspectives were considered, but these processes ultimately rely on subjective decisions.

This study relied on participants self‐identifying as experiencing socioeconomic disadvantage. As detailed in the methods, this approach was considered to offer some advantages. However, it relied on potential participants' making a subjective judgement in response to a question that some initially had difficulty understanding. Correspondingly, it would have been valuable to collect at least some objective data on participants' socioeconomic status to validate the self‐identification.

### 4.2 Implications for Practice and Future Research

This study was Phase 1 of a larger project (Figure 1). Phase 2 involved recruiting and training peer mentors. Phase 3 is exploring the feasibility and perceived usefulness of this study's adapted OA peer mentorship intervention. This represents a key next step in determining whether the adaptions have achieved their aims. The adapted OA peer mentorship intervention is a promising approach for reducing demands on healthcare services as it would be delivered by trained volunteers. It could be particularly accessible and inclusive for people experiencing socioeconomic disadvantage due to the remote delivery format and potential benefits of peer support, such as a sense of connection [16, 43].

A review investigating the effectiveness of self‐management interventions for long‐term conditions in people experiencing socioeconomic deprivation highlighted that delivery by peers may be beneficial [44]. The findings of this study suggest that having a peer with lived experience of the same condition is likely to be key. While the project team agreed at the start of the project that it would be important to recruit peer mentors who consider themselves to be experiencing socioeconomic disadvantage, this was not explicitly highlighted in the focus groups or interviews, so further research exploring this would be valuable.

Wider learning from this study could be applied to other peer mentorship and self‐management interventions. For example, this study's findings suggest it may be helpful to involve previously trained peer mentors as facilitators when delivering training to new peer mentors. This study has demonstrated how behavioural analysis tables can be used to map barriers and enablers to original intervention features or adaptations, which could applied in future adaptation studies.

## 5 Conclusion

This study rigorously and systematically adapted an in‐person OA peer mentorship intervention for remote delivery to people experiencing socioeconomic disadvantage. Given the potential accessibility and scalability of remote interventions, and the substantial health inequalities faced by people experiencing socioeconomic disadvantage, this could ultimately help to advance health equity. The multi‐method approach resulted in a larger number of adaptations being made than would have been the case if a single approach had been used. Including diverse partners was key to identifying adaptions required for people with different needs and preferences. This approach is likely to have optimised the chances that the adapted OA intervention will be feasible and useful in practice.

## Author Contributions

**Anna M. Anderson:** conceptualisation, formal analysis, funding acquisition, investigation, methodology, visualisation, writing – original draft preparation, writing – review and editing. **Elizabeth Lavender:** conceptualisation, formal analysis, funding acquisition, investigation, methodology, project administration, writing – original draft preparation, writing – review and editing. **Samantha Mason:** formal analysis, writing – original draft preparation, writing – review and editing. **Linda Eckersley:** conceptualisation, funding acquisition, methodology, writing – review and editing. **Susan Barry:** conceptualisation, funding acquisition, methodology, writing – review and editing. **Amrit Daffu‐O'Reilly:** formal analysis, investigation, project administration, writing – review and editing. **Heidi Green:** conceptualisation, funding acquisition, methodology, writing – review and editing. **Mark Conner:** conceptualisation, funding acquisition, methodology, validation, writing – review and editing. **Gretl A. McHugh:** conceptualisation, formal analysis, funding acquisition, methodology, supervision, writing – original draft preparation, writing – review and editing.

## Ethics Statement

Ethical approval for was gained from the South Birmingham Research Ethics Committee (23/WM/0108). All participants provided informed consent before participating. The project was performed in accordance with all relevant guidelines and regulations, including the Declaration of Helsinki.

## Conflicts of Interest

The authors declare no conflicts of interest.

## Supporting information

Supporting File 1: Image descriptions of the figures.

Supporting File 2: Socioeconomic disadvantage examples.

Supporting File 3: Topic Guide prompt questions.

Supporting File 4: Potential adaptations.

Supporting File 5: Key behaviour change techniques included in the adapted intervention.

## Data Availability

The data that supports the findings of this study are available in the supporting material of this article where appropriate. Additional data supporting the findings of this study are available on request from the corresponding author but are not publicly available due to privacy or ethical restrictions.
