# Supplementary material for: Adapting an Osteoarthritis Peer Mentorship Intervention for Remote Delivery to People Experiencing Socioeconomic Disadvantage: A Multi‐Method Approach
Source: Health Expect. 2025 Apr 1;28(2):e70245. doi: 10.1111/hex.70245 (PMC11959151; doi:10.1111/hex.70245)
Supplement: Supplementary file 1 — Supporting File 1: Image descriptions of the figures. [file HEX-28-e70245-s001.docx]

Supplementary File 1: Image descriptions of the figures

# Figure 1: Project flow chart image description

Project flow chart showing the cross-cutting principle and steps quoted from the ADAPT guidance (1), the osteoarthritis (OA) peer mentorship intervention adaptation phases, and the project team and groups.

## ADAPT guidance

Cross-cutting principle: Form an adaptation team of diverse stakeholders.

Step 1: Assess the rationale for intervention and consider intervention-context fit.

Step 2: Plan and undertake adaptations.

Step 3: Plan and undertake piloting and evaluation.

Step 4: Implement and maintain the adapted intervention at scale.

## OA peer mentorship intervention adaptation

Phase 1: Intervention development (mapped to ADAPT steps 1 and 2)

- Phase 1a: Exploring barriers and enablers
- Phase 1b: Developing and finalising the adapted intervention

Phase 2: Intervention set-up (mapped to ADAPT step 2)

- Recruiting and training peer mentors

Phase 3: Process evaluation (mapped to ADAPT step 3)

- Delivering and evaluating the adapted intervention
- Stakeholder discussion forum

Potential future work (mapped to ADAPT steps 3 and 4)

- Randomised controlled trial, implementation, and maintenance of the adapted intervention.

## Project team and groups

- Project team: two Patient and Public Involvement (PPI) members, seven professional members. The professional study team members included six university-based researchers and a Director of Health Equity.
- Project Advisory Group: three PPI members, nine professional members. The Project Advisory Group professional members included an independent chair, a community organisation representative, four clinical and academic experts, one co-production and community engagement expert, and two project funder representatives.
- PPI group: two study team PPI members, three Project Advisory Group PPI members, six additional PPI members.

# Figure 2: Participant flow chart image description

Phase 1a participant flow chart showing the reasons for exclusion at each stage.

Eighty-nine individuals expressed interest, of whom 53 were not screened due to the following reasons:

- Not contactable = 6
- Suspected imposters = 43
- Did not meet purposive selection criteria = 2
- Enquired too late = 1
- Did not answer all screening questions = 1

Thirty-six individuals were screened, of whom five were not eligible due to the following reasons:

- No experiencing socioeconomic disadvantage (SED) = 3
- No formal OA diagnosis = 2

Thirty-one individuals were eligible, of whom seven did not consent due to the following reasons:

- Decided to become a peer mentor = 1
- Did not complete consent form = 6

Twenty-four individuals consented, of who four did not participate due to the following reasons:

- Withdrew after consent = 3
- Lost contact after consent = 1

Twenty individuals participated.

# Figure 3: Logic model image description

Logic model of the adapted OA peer mentorship intervention summarising the problem for people with OA and SED, adapted OA peer mentorship intervention, proposed responses and mediators, and outcomes for people with OA and SED.

## Problem for people with OA and SED

Each of the following problems links to the next:

- Increased prevalence and burden of OA among people experiencing SED.
- Insufficient self-management support tailored to the needs of people with SED.
- Limited use of self-management strategies.
- High levels of pain, functional limitations, and psychological distress.
- Low health-related quality of life.

## Adapted OA peer mentorship intervention

Six one-hour remote peer mentorship sessions covering six core topics and individually selected optional topics.

Flexible delivery options and support

- Telephone or videoconferencing platform of choice
- Loan digital device and Wi-Fi support funds if required
- Reassurance about the remote format and digital coaching/support if required
- Flexibility with timing of sessions
- One-to-one or small group sessions
- Same gender matching if preferred
- Mentor who speaks same language/interpreter present if required

Core topics

- Self-managing OA
- Setting realistic goals
- Getting active, staying active
- Eating well, feeling well
- Activity pacing in practice
- Managing pain

Optional topics

- Getting motivated
- Relaxing and sleeping
- Communicating well
- Getting connected
- Having a joint replaced
- Getting support for work

## Proposed responses and mediators

Each of the following responses/mediators links to the next:

- Increased capability, opportunity, and motivation to attend remote peer mentorship sessions.
- Attendance at remote OA peer mentorship sessions.
- Increased capability, opportunity, and motivation to use self-management strategies.
- Increased use of self-management strategies such as:
  - Setting/reviewing goals
  - Performing exercises
  - Being physically active
  - Eating a healthy diet
  - Pacing activities
  - Using pain management strategies

## Outcomes for people with OA and SED

Improved pain, function and psychological well-being, linking to higher health-related quality of life.

Potential wider benefits e.g., reduced digital-related inequities.

# References

1. Moore G, Campbell M, Copeland L, Craig P, Movsisyan A, Hoddinott P, et al. Adapting interventions to new contexts—the ADAPT guidance. BMJ. 2021;374:n1679.
