# Supplementary material for: Adapting an Osteoarthritis Peer Mentorship Intervention for Remote Delivery to People Experiencing Socioeconomic Disadvantage: A Multi‐Method Approach
Source: Health Expect. 2025 Apr 1;28(2):e70245. doi: 10.1111/hex.70245 (PMC11959151; doi:10.1111/hex.70245)
Supplement: Supplementary file 2 — Supporting File 2: Socioeconomic disadvantage examples. [file HEX-28-e70245-s002.docx]

Supplementary File 2: Socioeconomic disadvantage examples

If potential participants had difficulty understanding the screening question about socioeconomic disadvantage, the following examples were provided:

- A person who is unhappy with their living conditions, for example, quality of housing, area, neighbours etc., but is unable to improve or move away from this because of their financial situation.
- A person may be unhappy with their job or feels they are not paid enough but is unable to secure another job because of their education levels.
- A person may experience loneliness because of where they live and may not be able to get to places to socialise with others because of finances, health, travel ability etc.
