# Supplementary material for: Adapting an Osteoarthritis Peer Mentorship Intervention for Remote Delivery to People Experiencing Socioeconomic Disadvantage: A Multi‐Method Approach
Source: Health Expect. 2025 Apr 1;28(2):e70245. doi: 10.1111/hex.70245 (PMC11959151; doi:10.1111/hex.70245)
Supplement: Supplementary file 3 — Supporting File 3: Topic Guide prompt questions. [file HEX-28-e70245-s004.docx]

Supplementary File 3: Topic guide prompt questions

# A: Self-management

1. In what ways do you manage/self-manage your osteoarthritis? How does that help you?
2. What prevents you from managing your osteoarthritis/ what makes it more difficult?
3. What might help you manage your osteoarthritis better?

# B: Information

1. How would you find out about managing your osteoarthritis?
2. How useful are the sources of information you have used to find out about osteoarthritis?
3. What, if any, difficulties have you had accessing the support you need?
4. What would you like to know more about?
5. Thinking about your osteoarthritis what type of support would be most helpful to you?
6. What type of support would be least helpful to you?

# C: Peer mentorship

1. What does the term peer mentor mean to you?
2. How do you think a peer mentor could support someone with osteoarthritis?
3. In what ways could a peer mentor help you manage your osteoarthritis better?
4. How would you feel about being supported by a peer mentor?
5. What would an ideal programme of peer mentorship for people with osteoarthritis look like?

# D: Experience of using the Internet/videoconferencing

1. Have you ever had a remote consultation or health support with a GP or other health professional? Was this consultation by telephone or videoconferencing?
2. Can you tell us about this? What was good / less good about it? What would have made it better?
3. Overall how confident would you feel about having remote health support?
4. Thinking about our discussion of peer mentorship support, how would you feel about receiving remote support (online or by telephone) to help you manage your osteoarthritis?

# E: General

1. Is there anything else that anyone would like to share or discuss further?
