# Supplementary material for: Adapting an Osteoarthritis Peer Mentorship Intervention for Remote Delivery to People Experiencing Socioeconomic Disadvantage: A Multi‐Method Approach
Source: Health Expect. 2025 Apr 1;28(2):e70245. doi: 10.1111/hex.70245 (PMC11959151; doi:10.1111/hex.70245)
Supplement: Supplementary file 4 — Supporting File 4: Potential adaptations. [file HEX-28-e70245-s005.docx]

Supplementary File 4: Potential adaptations

# Table S1: Suggested adaptations that were not addressed

| Source of suggestion | Suggested adaptation | Rationale for not making the adaptation |
| --- | --- | --- |
| Participatory workshops | Change ‘Relaxing and sleeping well’ to a core topic | - This change was suggested by one workshop attendee only. - The project team agreed that ‘Relaxing and sleeping well’ is an important topic, but the other six topics proposed as core are more in keeping with recommendations from wider evidence. - Mentees can still cover ‘Relaxing and sleeping well’ if they wish even if it is kept as an optional topic. |
|  | Include exercise videos to support mentees to learn and practice the exercises | - The mentee handouts already provide step-by-step written instructions and photographs of the exercises, and the feasibility trial of the in-person OA peer mentorship intervention (1) and feedback from some participatory workshop attendees suggested that would be sufficient. - Funds for creating videos were not available. - Viewing online videos might be difficult or impossible for some people, such as people who are not confident in using the Internet. |
|  | Offer peer mentors and mentees an introductory session with the research team or a group meet-up prior to the mentorship sessions to help build rapport | - This would add a seventh session to the programme, which would not be feasible within the project timescale and budget. - A member of the research team will be present at the start of the first mentorship session to help with building rapport. |
| Peer mentorship delivery practice runs | Include a picture of portion sizes in the ‘Eating well, feeling well’ core topic | - The Eat Well plate is already included in the mentee handouts and appropriate portion sizes vary between individuals and food types. |
|  | Adapt the core topics’ Self-managing osteoarthritis’ and ‘Setting realistic goals’ as they come across as a little ‘academic’ in style. | - Other feedback suggested the content is appropriate and it is important to ensure the key points about these topics are addressed. |
|  | Adapt the mentorship programme to encourage mentees to choose optional topics in weeks 1 and 2. | - The core topics and optional topics are based on the previous OA peer mentorship intervention and NICE OA guideline (2), so covering core topics in weeks 1 and 2 remains a priority. |
|  | Advise mentees to use an upright chair with arms to help with support and stability when exercising | - Mentees may not have a chair with arms, and it is adequate to recommend using a secure upright chair in the safety checklist. |
|  | Provide guidance on positioning a smartphone for exercising | - It is not possible to predict how this could work due to the peer mentors’ and mentees’ differing circumstances, and some may feel uncomfortable practising exercises in front of another person. - The ‘Getting active, staying active’ mentee handout includes photographs and guidance on how to perform the exercises. |
|  | Start the mentorship support sessions in the winter months as these can be ‘low mood’ months for some people | - The mentorship support sessions cannot be started in the winter months due to the study timelines. |

NICE, National Institute for Health and Care Excellence; OA, osteoarthritis.

# Table S2: Rationale for adaptations to the peer mentorship programme

| Area | Adaptation | Rationale for making the adaptation |
| --- | --- | --- |
| Peer mentorship programme delivery | Offer six support sessions | - A mean of six support sessions were provided during the feasibility trial of the in-person OA peer mentorship intervention (1). |
|  | Offer the option to hold mentorship sessions via telephone or videoconferencing | - ‘Low confidence in ability to make videoconferencing calls’, ‘Concerns about a remote format’ and ‘Feeling fed up with or stressed by video calls’ were identified as barriers in Phase 1a, while ‘Believing a remote format would be appropriate or advantageous’ was identified as an enabler. - The project team agreed that this adaptation is important for inclusivity. |
|  | Offer flexibility with the choice of videoconferencing platform | - ‘Experience of making video calls with a specific videoconferencing platform’ was identified as an enabler in Phase 1a. - The participatory workshop attendees felt mentees and peer mentors need to feel confident with the platform they use, and some individuals may be more experienced with a particular platform(s). - Feedback from the mentorship delivery practice runs suggested using a familiar platform for peer mentorship support sessions would encourage engagement. |
|  | Ensure the peer mentors who deliver the programme consider themselves to be experiencing socioeconomic disadvantage | - The project team agreed that this adaptation is an important element of peer support for people experiencing socioeconomic disadvantage. |
|  | Offer the option of having a peer mentor who speaks the same language or an interpreter present | - ‘Peer mentor who speaks the same language or an interpreter present’ was identified as an enabler in Phase 1a. - Based on the project team’s experience and wider literature, offering the intervention in different languages is important for inclusivity. - The participatory workshop attendees felt it is important to consider the needs of people whose first language is not English and take a flexible approach to this depending on the languages spoken by the mentees and peer mentors. |
|  | Offer the option of having mentorship in small groups (rather than one-to-one only) if feasible | - ‘Believing a group format would be advantageous’ and ‘Believing a one-to-one format would be advantageous’ were identified as a barrier and enabler respectively in Phase 1a. - The participatory workshop attendees felt it is important to have flexibility with the format depending on the preferences of the mentees and peer mentors. |
|  | Offer mentees reassurance about the remote format and digital coaching/support | - ‘Low confidence in ability to make videoconferencing calls’, ‘Concerns about a remote format’ and ‘Feeling fed up with or stressed by video calls’ were identified as barriers in Phase 1a, while ‘Believing a remote format would be appropriate or advantageous’ was identified as an enabler. - The project team and participatory workshop attendees felt this adaptation is important to support digital inclusion. - Feedback from the mentorship delivery practice runs emphasised some people may need support to use a videoconferencing platform. |
|  | Offer support for mentees with additional communication needs (e.g. for people who are Deaf or hard of hearing) | - The project team agreed that this adaptation is important for inclusivity. - The participatory workshop attendees also suggested this is important for inclusivity. |
|  | Provide mentees with printed and electronic copies of the mentee handouts prior to their first support session | - The project team and participatory workshop attendees felt this adaptation is important to allow mentees to become familiar with the handouts prior to the first support session and meet people’s individual preferences. - Feedback from the mentorship delivery practice runs emphasised this adaptation is important. |
| Peer mentorship programme content and resources | Adapt the wording of the mentee recruitment materials to clarify the concept of peer mentorship | - ‘Not knowing what peer mentorship is’ and ‘Knowing what peer mentorship is’ were identified as a barrier and enabler respectively in Phase 1a. |
|  | Split the peer mentor resource pack and mentee handouts into two separate documents with the acknowledgements at the end of each document | - The project team agreed that this change is important to improve the clarity of the resources, reduce the amount of text on each page, and reduce the overall length of the resource pack. |
|  | Change the images to maximise diversity and relevance for peer mentors and participants | - The project team agreed that this adaptation is important for inclusivity to make the resources more widely relatable. |
|  | Add guidance to the resource pack on providing mentorship support remotely and amend the document’s structure accordingly | - The project team agreed that this adaptation is important to ensure peer mentors can feasibly deliver the mentorship sessions. |
|  | Adapt the wording and formatting of the resource pack and mentee handouts to improve readability, clarity, and accessibility | - ‘Difficulty understanding online self-management information’ and ‘Understanding and acting on self-management information’ were identified as a barrier and enabler respectively in Phase 1a. - The project team agreed that this adaptation is important for inclusivity and would help to reduce the overall length of the documents. - The participatory workshop attendees highlighted it is important to ensure the exercise instructions are easy to understand. |
|  | Adapt the content of the resource pack and handouts to ensure it aligns with the current NICE OA guideline (2) and relevant evidence | - A new NICE OA guideline was published after the development of the in-person OA peer mentorship intervention (2). |
|  | Change ‘Managing pain’ to a core topic | - ‘Believing that pain management medications are ineffective’ was identified as a barrier in Phase 1a. - Most peer mentor/mentee dyads in the feasibility trial of the in-person OA peer mentorship intervention covered the ‘Managing pain’ topic (1) and the project team agreed this topic should be prioritised. |
|  | Change ‘Eating well, feeling well’ to a core topic | - The current NICE OA guideline recommends weight management as a core treatment for OA (2) and the project team agreed that healthy eating and weight management should be prioritised. |
|  | Change ‘Getting connected’ to an optional topic | - ‘Limitations with support from friends, family, and groups’ and ‘Support from friends, family, and groups’ were identified as a barrier and enabler respectively in Phase 1a. - Most peer mentor/mentee dyads in feasibility trial of the in-person OA peer mentorship intervention did not cover the ‘Getting connected’ topic (1) and the project team agreed the topic is not a priority for all. |
|  | Combine the ‘Building muscle strength’ core topic and ‘Building an active lifestyle’ optional topic into a single ‘Getting active, staying active’ core topic | - The project team agreed that this adaptation is important so that both therapeutic exercise and physical activity are prioritised, and mentees are encouraged to focus on functional exercises (rather than exercises in lying/sitting) where possible. |
|  | Combine the ‘Breathing and relaxation’ and ‘Sleep quality’ optional topics into one ‘Sleeping and resting’ optional topic | - The project team agreed this adaptation is important to help keep the number of optional topics manageable. |
|  | Add ‘Having a joint replaced’ as an optional topic | - A PPI member of the project team member felt this is an important topic to offer to mentees, and the other team members and participatory workshop attendees agreed. - The feedback on the grant application highlighted that peer mentorship could make a contribution in the context of long waiting lists for surgery. - Joint replacement is a relatively common procedure for people with OA. |
|  | Add ‘Getting support for work’ as an optional topic | - The project team agreed that this is an important topic to offer to mentees given that some mentees may be working/aiming to return to work and people with OA may benefit from support with returning to and staying in work. |
|  | Adapt the ‘Setting realistic goals’ topic in the resource pack to provide clearer guidance on how to set action plans and include different goal examples | - The project team agreed that it would be helpful to provide clearer guidance on setting goals and action plans. |
|  | Add guidance on weight management to the ‘Eating well, feeling well’ topic in the resource pack and mentee handouts, including guidance for peer mentors on how to approach the topic sensitively | - The current NICE OA guideline recommends weight management as a core treatment for OA (2) and the project team and participatory workshop attendees agreed that weight management is important to cover, but needs to be approached sensitively. |
|  | Offer culturally adapted versions of the ‘Eatwell guide’ in the mentee handouts | - The participatory workshop attendees highlighted it is important to have guidance and resources for mentees from other cultures. - The traditional western ‘Eatwell’ plate may not be appropriate to use with people from certain cultures. |
|  | Adapt the ‘Getting active, staying active’ topic text in the resource pack to emphasise that physical activity is usually helpful not harmful | - The participatory workshop attendees raised some concerns about physical activity being harmful. |
|  | Adapt the text in the ‘Getting active, staying active’ topic in the mentee handouts to highlight that the activity plan can be undertaken in any comfortable clothing | - The participatory workshop attendees highlighted that the model in the photographs demonstrating the exercises is dressed in exercise clothes and this may put mentees off due to believing specific clothes are needed to exercise. - The photographs of the model in exercise clothes are useful for clearly demonstrating the position of the body when exercising |
|  | Provide the Versus Arthritis ‘Let’s Move Tracker’ as an extra document in the ‘Getting active, staying active’ topic | - The project team agreed that the ‘Let’s Move Tracker’ would be useful to provide a simple way for mentees to record their activity sessions. |
|  | Add a safety checklist to the ‘Getting active, staying active’ topic in the mentee handouts | - The mentorship delivery practice run feedback suggested this adaptation would be helpful for mentees/peer mentors to refer to. |
|  | Include the Versus Arthritis booklets ‘Footcare and footwear’ as an extra document with the handouts if indicated | - ‘Use of aids or resources (including cushioned trainers) to self-manage’ was identified as an enabler in Phase 1a. - The mentorship delivery practice run feedback suggested caution is needed when suggesting outdoor activities (particularly in the winter months) due to the increased risk of falls, so providing information on appropriate footwear may be helpful. |
|  | Include the Versus Arthritis booklet ‘Sleep and arthritis’ as an extra document with the handouts if indicated | - The mentorship delivery practice run feedback suggested that providing the ‘Sleep and arthritis’ booklet may be helpful for some mentees. |
|  | Include the Versus Arthritis ‘Eating well with arthritis’ booklet as an extra document with the mentee handouts for all mentees | - The mentorship delivery practice run feedback suggested more detail about healthy eating may be required. |
|  | Add a reminder to regularly recap on previously covered topics to the ‘Delivering support sessions’ section of the resource pack | - The participatory workshop attendees suggested peer mentors re-cap on previous sessions with their mentees at the start of the next session. Continuity and reminders are important when embarking upon behaviour change and a re-cap is often useful. |
|  | Remove the links to local support organisations from the ‘Getting connected’ topic in the mentee handouts | - The project team agreed that the local links would not be relevant given that the mentorship may be delivered to individuals from anywhere in the UK. |
|  | Add extra links to national support organisations to the ‘Getting connected’ topic in the mentee handouts, including links for local group search functions, and highlight that mentees could search for other opportunities | - ‘Difficulty finding relevant self-management information online’ was identified as a barrier in Phase 1a. - The project team agreed that this adaptation is important due to the links to local support organisations being removed and to assist mentees with finding options for follow-on support once the peer mentorship has ended. - The participatory workshop attendees felt it would be helpful to compile a list of additional services to signpost mentees to for additional and/or ongoing support. |
|  | Add extra pages for mentees to make notes at the end of the mentee handouts | - Feedback from the mentorship delivery practice runs suggested including space for notes in each section of the mentee handouts would be useful. - The project team agreed adding notes at the end of the handouts would be the best option to prevent the handouts becoming too lengthy. |
|  | Offer mentees a loan digital device and Wi-Fi support funds | - ‘Lack of access to a digital device and adequate internet connection’ was identified as a barrier in Phase 1a. - The project team agreed that this adaptation is important for digital inclusion. - Feedback from the mentorship delivery practice runs suggested there needs to be flexibility with the devices used during the mentorship sessions, so some mentees may require a loan digital device and support funds. |

NICE, National Institute for Health and Care Excellence; OA, osteoarthritis.

# Table S3: Rationale for adaptations to the peer mentor training

| Area | Adaptation | Rationale for making the adaptation |
| --- | --- | --- |
| Peer mentor training programme delivery | Deliver the training remotely via videoconferencing | - The project team agreed that this adaptation is important due to the peer mentors being recruited from across the UK. |
|  | Offer peer mentors digital coaching/support | - The project team and participatory workshop attendees felt this adaptation is important to support digital inclusion. - Feedback from the mentorship delivery practice runs suggested some peer mentors may benefit from training on videoconferencing functions such as screen sharing and mute etc. |
|  | Deliver the training over two non-consecutive days | - The project team found this was helpful during the feasibility trial of the in-person OA peer mentorship intervention (1). - The participatory workshop attendees felt this would be helpful as online learning may be challenging/less engaging and concentration levels may vary. Holding the training over non-consecutive days would allow attendees to have a rest between training days and help consolidate the information from the first day. |
|  | Incorporate more breaks into the training | - The participatory workshop attendees suggested regular breaks and shorter sessions may help to reduce fatigue and keep mentors alert and engaged throughout the training programme. |
|  | Use breakout rooms to provide opportunities for small group discussions and role play | - The participatory workshop attendees felt this adaptation would help engagement with interactive activities. |
|  | Include peer mentors from the previous study as facilitators for role play and discussion | - The project team and participatory workshop attendees felt it is important for peer mentors to feel confident with knowing how to deal with potential problems prior to delivering support sessions and learn from the experience of previous peer mentors. |
|  | Provide peer mentors with printed and electronic copies of the resource pack and mentee handouts, and the administrative documentation (e.g. confidentiality form), prior to the training | - The project team and participatory workshop attendees felt this adaptation is important to allow peer mentors to become familiar with the handouts prior to the training. - Providing the documents in advance of the training reduces the work burden on the day of the training. - Printed documents are useful as a backup in case of technical or access issues and may be preferred by some individuals. - Feedback from the mentorship delivery practice runs emphasised this adaptation is important. |
|  | Provide peer mentors with brief verbal information about their mentees’ self-management needs prior to the introductory session (with the mentees’ consent) | - The participatory workshop attendees and feedback from the mentorship delivery practice run suggested it would be helpful to provide a brief written profile of mentees to help with the introduction. However, to meet data protection/confidentiality requirements, the project team agreed that providing brief verbal information would be more appropriate. |
| Peer mentor training programme content | Include training on how provide remote support with the home exercise plan and tailor the exercise plan for mentees who have restricted mobility and/or use mobility aids | - The participatory workshop attendees felt it is important to train the peer mentors to be knowledgeable about and competent with the exercises, including how to adapt them for mentees who have restricted mobility and/or use mobility aids. |
|  | Include additional emphasis that physical activity is usually helpful not harmful | - The participatory workshop attendees raised some concerns about physical activity being harmful. |
|  | Include a suggestion about putting a smartphone (if used) on speaker during the mentorship sessions | - Feedback from the mentorship delivery practice runs suggested it may be helpful to use a smartphone on speaker during the mentorship sessions to enable easier interaction and use of resources |
|  | Include information about considering falls risk when recommending outdoor activities | - The mentorship delivery practice run feedback suggested caution is needed when suggesting outdoor activities (particularly in the winter months) due to the increased risk of falls. |
|  | Encourage peer mentors to ensure they regularly recap on previously covered topics during the mentorship sessions | - The participatory workshop attendees suggested peer mentors re-cap on previous sessions with their mentees at the start of the next session. - Continuity and reminders are important when embarking upon behaviour change and a re-cap is often useful. |
|  | Offer peer mentors a loan digital device and Wi-Fi support funds | - The project team agreed that this adaptation is important for digital inclusion. - Feedback from the mentorship delivery practice runs suggested there needs to be flexibility with the devices used during the mentorship sessions, and some mentees may require a loan digital device and support funds. |

UK, United Kingdom.

# References

1. Anderson AM, Lavender EC, Dusabe-Richards E, Mebrahtu TF, McGowan L, Conaghan PG, et al. Peer mentorship to improve self-management of hip and knee osteoarthritis: a randomised feasibility trial. BMJ Open. 2021;11(7):e045389.

2. National Institute for Health and Care Excellence (NICE). Osteoarthritis in over 16s: diagnosis and management (NICE guideline [NG226]). 2022.
