# Supplementary material for: Adapting an Osteoarthritis Peer Mentorship Intervention for Remote Delivery to People Experiencing Socioeconomic Disadvantage: A Multi‐Method Approach
Source: Health Expect. 2025 Apr 1;28(2):e70245. doi: 10.1111/hex.70245 (PMC11959151; doi:10.1111/hex.70245)
Supplement: Supplementary file 5 — Supporting File 5: Key behaviour change techniques included in the adapted intervention. [file HEX-28-e70245-s003.docx]

Supplementary File 5: Key behaviour change techniques included in the finalised adapted osteoarthritis peer mentorship intervention

| **Behaviour change technique^a^** | **Example of implementation** |
| --- | --- |
| 1.1 Goal setting (behaviour) | The peer mentor supports the mentee to set a goal of walking to meet a friend for coffee at their house twice weekly. |
| 1.2 Problem solving | The peer mentor supports the mentee to analyse barriers to pacing their activities and identify strategies to help overcome the barriers. |
| 1.4 Action planning | The peer mentor supports the mentee to plan to practise heel raises while waiting for the kettle to boil after lunch each day. |
| 1.5 Review behaviour goal(s) | The peer mentor and mentee review the mentee’s goal of not drinking any caffeinated drinks after 5.00pm and adapt it if necessary. |
| 2.2 Feedback on behaviour | The peer mentor provides the mentee with feedback on their strengthening exercise technique during their peer mentorship session. |
| 3.1 Social support (unspecified) | The peer mentor provides information about a local walking football group. |
| 3.3 Social support (emotional) | The peer mentor provides emotional support to help the participant to go for a walk even though they feel anxious about falling. |
| 4.1 Instruction on how to perform the behaviour | The peer mentor advises the participant how to perform a relaxation technique. |
| 5.1 Information about health consequences | The peer mentor explains that losing weight can help improve symptoms of osteoarthritis. |
| 5.6 Information about emotional consequences | The peer mentor explains that using conscious breathing techniques can help people feel calmer. |
| 6.1 Demonstration of the behaviour | The peer mentor demonstrates how to perform chair-based exercises themselves via screensharing and/or by encouraging the mentee to look at the exercise demonstration photographs in the mentee handouts. |
| 6.2 Social comparison | The peer mentor draws attention to how they are able to do yoga despite their osteoarthritis. |
| 8.1 Behavioural practice/rehearsal | The peer mentor prompts the mentee to practise being assertive in preparation for their doctor’s appointment. |
| 8.3 Habit formation | The peer mentor prompts the mentee to carry out an exercise routine in their bedroom after they have had a shower each morning. |
| 8.7 Graded tasks | The peer mentor supports the mentee to walk 2km by advising them to start with walking 1km and then add an extra 100m each week. |
| 9.1 Credible source | The peer mentor provides communication in favour of eating five portions of fruit and vegetables per day e.g., by reviewing the Versus Arthritis ‘Eating well with arthritis’ booklet. |
| 10.4 Social reward | The peer mentor praises the mentee for progressing towards their goal of getting up at 8.30am each morning. |
| 12.1 Restructuring the physical environment | The peer mentor and mentee agree to hold the mentorship sessions via telephone rather than videoconferencing |
| 12.2 Restructuring the social environment | An interpreter is present to enable the mentorship sessions to be held in the mentee’s preferred language |
| 12.5 Adding objects to the environment | The project team provide a loan digital device to enable the mentee to take part in the mentorship sessions via videoconferencing |
| 15.1 Verbal persuasion about capability | A project team member provides digital coaching/support, including telling the mentee they can successfully make videoconferencing calls despite the mentee reporting low confidence in their ability to make videoconferencing calls |

^a^ Behaviour change techniques are coded using the Behaviour Change Technique Taxonomy version 1 (1).

Only key behaviour change techniques are listed. Peer mentors could incorporate additional behaviour change techniques as appropriate to the mentee’s individual needs and goals.

This table is a modified version of Supplementary Table 3 in Anderson et al (2) created under the terms of the Creative Commons Attribution 4.0 Unported (CC BY 4.0) license (<https://creativecommons.org/licenses/by/4.0/>).

# References

1. Michie S, Richardson M, Johnston M, Abraham C, Francis J, Hardeman W, et al. The Behavior Change Technique Taxonomy (v1) of 93 hierarchically clustered techniques: building an international consensus for the reporting of behavior change interventions. Ann Behav Med. 2013;46(1):81-95.

2. Anderson AM, Lavender EC, Dusabe-Richards E, Mebrahtu TF, McGowan L, Conaghan PG, et al. Peer mentorship to improve self-management of hip and knee osteoarthritis: a randomised feasibility trial. BMJ Open. 2021;11(7):e045389.
